# Thiol-free reducing agents in electrophoretic separations and FASP proteolytic digestions for the analysis of metal-binding proteins

**DOI:** 10.1016/j.mex.2014.08.003

**Published:** 2014-08-20

**Authors:** I. Moraleja, E. Moreno-Gordaliza, M.L. Mena, M.M. Gómez-Gómez

**Affiliations:** a Analytical Chemistry Department, Universidad Complutense de Madrid, Avda. Complutense s/n, 28040 Madrid, Spain; b Division of Analytical Biosciences, LACDR, Universiteit Leiden, P.O. Box 9502, 2300 RA Leiden, The Netherlands

**Keywords:** SDS-PAGE, OFFGEL-IEF, FASP proteolytic digestion, Metal-binding proteins, OFFGEL-IEF, FASP, ICP-MS, Thiol-free reductants, nLC–ESI-LTQ-MS/MS

## Abstract

The analysis of the complexes between metal-based chemotherapeutic drugs and proteins in biological samples, such as cisplatin or oxaliplatin, can be a challenge due to metal strong reactivity towards S-donor molecules such as dithiothreitol (DTT) or β-mercaptoethanol (BME), usually employed as reducing agents in electrophoretic separations and proteolytic digestions for LC–MS/MS analysis.

• This protocol describes the use of the thiol-free reducing trialkylphosphines, such as tributylphosphine (TBP) and tris(2-carboxyethyl)phosphine (TCEP) as suitable reagents for the preservation of the metal-protein complexes during OFFGEL-IEF and SDS-PAGE separations, respectively.
• Moreover, the filter-aided sample preparation (FASP) method is presented as an advantageous option to perform tryptic in-solution digestions of metal–protein complexes in combination with OFFGEL-IEF separations.
• The FASP procedure allows including previous reduction and alkylation steps in addition to proteolysis, ensuring the preservation of the metal–protein complexes. The limited time that proteins remain in contact with the reducing agent, either TBP or even DTT, during FASP could be a key factor for its extraordinary performance on the digestion of metal–protein complexes.

## Graphical abstract

**Figure:**
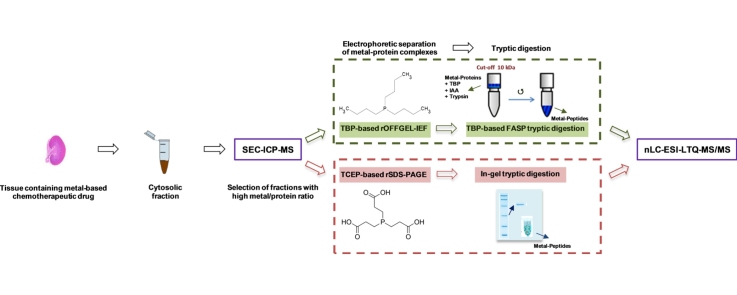

## Method details

Two protocols are detailed for the analysis of metal–protein complexes in biological samples, based on methods recently demonstrated for kidney cytosolic proteins from rats treated with platinum-based chemotherapeutic drugs. The described procedures include either TCEP-based rSDS-PAGE [1], or TBP-based rOFFGEL-IEF in combination with a FASP tryptic digestion [2], followed by identification by nLC–ESI-LTQ-MS/MS.

Prior to both protocols, a sample preparation step should be performed in order to obtain cytosolic protein fractions with the highest Pt to protein ratio from the metallodrug-exposed kidney by size exclusion chromatography (SEC)-ICP-MS.

### **Sample preparation**

#### Materials

• Buffer solution: 10 mM Tris–HCl (pH 7.4) containing 25 mM NaCl. Prepare the buffer with Milli-Q grade water.
• Protease inhibitor cocktail (Sigma–Aldrich).
• Electric drill capable of a minimum of 1000 r.p.m.
• Teflon–glass dounce homogenizer.
• Ultracentrifuge and 15 mL clear tubes, compatible with centrifugation at 15,000 × *g*.
• Ice buckets, Petri dish and a scalpel.
• Superdex™ 7510/300 GL column (GE Healthcare, separation range of 3–70 kDa)
• High-pressure quaternary gradient pump (Jasco PU-2089) equipped with an Injection valve (Rheodyne).
• Chromatographic conditions: Mobile phase: 10 mM Tris–NO_3_, 25 mM NaCl, pH 7.4; Flow rate: 0.8 mL min^−1^; Injection volume: 200 μL.
• Quick Start Bradford protein assay (Bio-Rad)
• Quadrupole ICP-MS equipped with a Meinhard nebulizer, a fassel torch, and an impact bead quartz spray chamber cooler by a Peltier system.
• Amicon Ultra-4 mL centrifugal filter (Millipore) with a nominal cut-off of 3 kDa.

#### Procedure

1. Place the kidney tissue (about 0.250 g) from a rat treated with a pharmacological dose of a metal-based chemotherapeutic drug, such as oxaliplatin, in a Petri dish on ice and mince the tissue with a scalpel.! Caution: Always wear laboratory gloves when handling and preparing solutions, samples, apparatus, etc. to prevent contamination of samples and solutions (i.e. skin keratins). This caution is to be extended to all the protocols described later.
2. Place the tissue in a tube and add 3 mL of pre-chilled buffer solution. To minimize proteolysis, add 12.5 μL of the protease inhibitor cocktail and always keep the tube on ice during steps 2–4.
3. Homogenize for a minimum of 15 min using a tight-fitting Teflon pestle attached to a power drill (set to >1000 r.p.m.) by slowly stroking the pestle up and down, taking 15 s per stroke and 30 s to complete a down and up cycle.
4. Inspect the homogenate; if intact tissue is still apparent, re-homogenize for an additional 1 min.! Caution: Wear safety glasses and a face shield, as the glass tube may shatter if too much pressure is applied or if the drill is moved at an angle other than vertical.
5. Decant the homogenate into an appropriate-size centrifuge tube and place into a pre-cooled rotor and centrifuge at 15,000 × *g* for 40 min.
6. Decant and save the cytosolic supernatant on an ice-bath.▴Critical step: all the previous preparative steps should be performed at 4 °C to minimize the risk of species degradation or transformation. Inert atmosphere is not needed. Possible species oxidation could be considered by setting oxidized methionines as variable modifications in Sequest searches.■Pause point: The supernatant can be kept frozen at −80 °C until required.
7. Inject the kidney cytosolic fraction through a 0.22 μm PVDF filter to the SEC column.
8. Collect fractions every minute (0.8 mL each).
9. Determine the Pt content in each fraction by ICP-MS (monitoring ^195^Pt isotope, channels per AMU: 10 and integration time: 0.6 ms), and the protein content by Bradford assay.
10. Identify the fractions with the highest Pt to protein ratio, which are selected for further analysis.
11. For their preconcentration and clean-up, the pool of ten identical selected SEC fractions was ultrafiltered through an Amicon Ultra-4 mL, 3 kDa cut-off filter. Collect the retentate.

## Protocol A: TCEP-based rSDS-PAGE

### Materials

• Bio-Rad's Laemmli sample buffer (LSB), containing Tris–HCl (62.5 mM, pH 6.8), glycerol (25%), SDS (2%) and bromophenol blue (0.01%) (Bio-Rad).
• Mini Protean^®^ Tetra Cell Electrophoresis System (Bio-Rad).
• Stacking gel for rSDS-PAGE was prepared at 3%, and resolving gels at 12.5% of polyacrylamide. Commercial precast gels can alternatively be used.
• Running buffer: contains Tris–HCl (25 mM, pH 8.3), glycine (192 mM) and SDS (0.1%). Prepare the buffer with Milli-Q grade water.
• Fixing solution: Milli-Q grade water/methanol/acetic acid (72.5/20/7.5).
• Colloidal Coomassie Blue: Bio-Safe™ Coomassie Stain (Bio-Rad).
• Trypsin Gold (MS grade, Promega).
• NanoLC system coupled to an ESI-LTQ-MS/MS instrument.
• Trap column (Reprosil pur C18, 3-μm particle size, 0.3 mm × 10 mm, 120 Å pore size, SGE).
• Microcapillary analytical column (Acclaim PepMap 100, C18, 3-μm particle size, 75 μm × 15 cm, 100 Å pore size, Dionex, LC Packings).
• Nano-bore emitter (O.D. 150 μm, I.D. 30 μm, Proxeon).
• Buffer A: 2% Mass Spec-grade acetonitrile, 0.1% formic acid in mass spec-grade water.
• Buffer B: 0.1% formic acid in mass spec-grade acetonitrile.
• Database search engine such as Sequest or Mascot (Matrix Science) for protein identification.

### Procedure

1. For subsequent SDS-PAGE separation, add 200 μL LSB to the preconcentrated cytosolic fraction (the retentate fraction from step 11 (Sample preparation procedure)) and vortex.
2. Heat at 95 °C for 1 min in a thermoblock.
3. Allow to cool down the sample, and once at room temperature (RT), add TCEP to a final concentration of 50 mM. In comparison to the conventional SDS-PAGE method [3], the heating time was shortened to 1 min and the reductant was added at a lower temperature (RT instead of 95 °C).
  ▴Critical step: avoid boiling samples with the reductant in order to preserve the integrity of the metal–protein complexes.
4. Load the sample (a total protein content of 50 μg) into the gel wells. Start electrophoresis at constant current (12 mA per gel until samples were stacked an then the current was increased to 20 mA per gel until the end of the separation).
5. Wash the gels with Milli-Q grade water for 20 min and incubate the gel in the fixing solution for 1 h in an oscillating shaker.
6. Stain the gel with colloidal Coomassie Blue for 1 h.
7. Wash the gel twice with 20 mL of Milli-Q grade water for 1 h per wash.
8. Excise protein bands from the gels with a scalpel.
9. Wash the gel slices for at least 1 h in 500 μL of 50 mM NH_4_HCO_3_. Discard the wash.
10. Wash the gel slices in 500 μL of 50% acetonitrile/50 mM NH_4_HCO_3_ with shaking for 1 h. Discard the wash. Cut the gel band into 1 mm^2^ pieces and transfer to a 500 μL Protein Lo-Bind tube (Eppendorf).
  ▴Critical step: Make sure that the gel slices stay wet with the wash solution to facilitate cutting and transfer.
11. Add 50 μL of acetonitrile to shrink the gel pieces. After 10–15 min, remove the solvent and dry the gel slices in a centrifugal evaporator.
12. Re-swell the gel pieces with 50 mM NH_4_HCO_3_ containing 12.5 ng/μL of trypsin and keep them in ice during reswelling (as required, typically add 10–20 μL). Once the gels have completely re-swollen, add 50 mM NH_4_HCO_3_ to cover the gel pieces (around 10–20 μL). Cap the tubes tightly and cover with parafilm to avoid evaporation. Incubate at 37 °C overnight (∼16 h) with gentle agitation using a thermomixer at 300 r.p.m.
  ▴Critical step: gel pieces need to stay wet during the digestion.
13. After completing the digestion, collect supernatants and transfer them to a Lo-Bind Eppendorf, keeping them at 4 °C. Next, extract the peptides remaining in the gel with 30 μL of 2% formic acid by vortexing, and incubate for 30 min at RT. Pool the extracted peptides with the original supernatant.
14. Finally, add 30 μL of a solution containing 50% acetonitrile and 0.1% formic acid, and incubate for another 30 min at RT. Pool the extraction solution with the previous ones, and evaporate samples in a vacuum centrifuge to dryness. Store the dry peptides at −80 °C until required, within a few months.
15. For protein identification, analysis by nLC–ESI-LTQ-MS/MS (or a similar LC–MS/MS platform) is proposed. Resuspend the dried peptides in 10 μL of buffer A and sonicate for 10 min in an ultrasonic bath. Then start the analysis as follows: Inject aliquots of 5 μL, using a 20 μL loop and a pick-up method, and load on a trap-column at a 3 μL min^−1^ flow rate using buffer A as mobile phase. Reverse the flow at 200 nL min^−1^ to elute and transfer the preconcentrated peptides to a reverse phase microcapillary analytical column. Elute peptides applying a three-step gradient: 5–15% B linear for 5 min, 15–40% B linear for 40 min and 40–80% B linear for another 15 min, holding the system at 80% B for 10 min. The end of the column was connected to a stainless steel nano-bore emitter for spraying and coupling with the LTQ. The spray voltage was set at 1.70 kV. Peptides were scanned and fragmented using a triple play scan method, consisting on acquisition of full enhanced MS scans in the positive ion mode, over the *m/z* range 400–1600, followed by zoom scans and further full enhanced MS/MS, acquired in profile mode, of the three most intense peaks in the full MS scan. CID activation of ions was applied in MS/MS experiments, with 35% relative collision energy and 30 ms activation time, being isolation width of the precursor ions set to 4. Dynamic exclusion was enabled with a repeat count of 1, using a 180 s exclusion duration window. For data analysis, spectra were assessed with the Xcalibur Qual Browser software (Thermo Scientific). MS/MS spectra search on NCBI protein databases using SEQUEST and MASCOT allowed the identification of proteins. The search was performed against a rat (*Rattus norvegicus*) NCBI database.

## Protocol B: TBP-based rOFFGEL-IEF in combination with a FASP tryptic digestion

### B1. TBP-based rOFFGEL-IEF

The following instructions assume the use of an Agilent 3100 OFFGEL Fractionator. Similar devices can also be used.

#### Materials

• Focusing buffer: contains urea 6.6 M, TBP 2.0 mM, ampholytes and 9.6% glycerol.
• Rehydration buffer: 0.96 mL of focusing buffer + 0.24 mL of Milli-Q water.
• Agilent 3100 OFFGEL Fractionator (Agilent Technologies) with a 24-well setup kit.
• IPG Strips 24 cm, pH 3–10 (store at −20 °C).
• Frames, cover seal, electrode pads and mineral oil (cover fluid).

#### Procedure

1. For subsequent OFFGEL-IEF fractionation, add 2.88 mL of focusing buffer and 0.72 mL of Milli-Q grade water (3.6 mL final volume) to the preconcentrated cytosolic fraction (the retentate fraction from step 11 (sample preparation procedure)) and vortex. With respect to the original OFFGEL protocol for protein separation [4], DTT was replaced by TBP.
  ▴Critical step: Do not add thiourea (used in the original OFFGEL protocol) to the focusing buffer, in order to preserve the integrity of the metal–protein complexes, such as cisplatin, as was earlier reported [5].
2. Place a pH 3–10 IPG strip in the system tray and rehydrate according to the manufacturer's instructions with 40 μL of rehydration buffer.
  ! Caution: Be careful not to touch the gel strip during the loading process.
3. Load 150 μL of sample in each well.
4. Start the fractionation as follows: focus the sample at 20 °C with a maximum current of 50 μA, and typical voltages ranging from 500 to 4000 V until 50 kV h^−1^ is reached after 24 h, approximately.
5. After the fractionation, slowly aspirate with a pipette the liquid phase from each well. Transfer each recovered fraction (volumes between 100 and 150 μL) to different 500 μL Protein Lo-Bind Eppendorf tubes.
  Pause point: The samples can be frozen at −80 °C until needed.

### B2. FASP procedure

OFFGEL-IEF fractions were trypsin-digested by FASP, as an alternative to the traditional in-solution tryptic digestion, as described as follows:

#### Materials

• Vivacon 0.5 mL spin filter (Sartorius Stedim Biotech) with a nominal cutoff of 10 kDa.
• UA solution: urea 8 M in NH_4_HCO_3_ 50 mM.
• UB solution: urea 1 M in NH_4_HCO_3_ 50 mM.
• TBP 10 mM in UA solution.
• Iodoacetamide (IAA) 420 mM in NH_4_HCO_3_ 50 mM.
• Trypsin Gold (MS grade, Promega).
• OMIX Tips C_18_, 100 μL (Agilent Technologies).
• NanoLC system coupled to an ESI-LTQ-MS/MS instrument.
• Buffer A: 2% Mass Spec-grade acetonitrile, 0.1% formic acid in mass spec-grade water.
• Buffer B: 0.1% formic acid in mass spec-grade acetonitrile.
• Database search engine such as Sequest or Mascot (Matrix Science) for protein identification.

#### Procedure

1. Load 100 μL of the OFFGEL-IEF fractions with a high amount of Pt (measured by ICP-MS and containing a total protein content of 250 μg) into a Vivacon spin filter.
2. Add 200 μL of UA solution and centrifuge at 14,000 × *g* for 15 min at 25 °C. Discard the filtrate. Repeat this step.
3. Dilute the retentate 1:1 with TBP 10 mM solution to a final concentration of TBP 5 mM, and incubate 30 min at room temperature for reducing the disulfide bonds of proteins. Centrifuge at 14,000 × *g* for 15 min at room temperature. Discard the filtrate.
  ▴Critical step: The use of TBP is a modification to the original FASP protocol [6,7]. However, the risk of metal-complexes loss is minimized even using DTT due to the fact that reagents employed along the process are subsequently eliminated by centrifugation before the following step.
4. Add IAA solution to a final concentration of 20 mM, and incubate 30 min at room temperature in the dark. Centrifuge at 14,000 × *g* for 15 min at room temperature. Discard the filtrate.
5. Add 100 μL of UB solution to the protein retentate as a clean-up step. Repeat this step twice. Discard the filtrate.
6. Add 5 μg of trypsin in 100 μL of UB solution to the retentate, and incubate the samples at 37 °C overnight.
7. Collect the peptides by centrifugation at 14,000 × *g* for 15 min at room temperature.
8. Add 30 μL of UB solution and centrifuge at 14,000 × *g* for 15 min at room temperature. Repeat this step three times, pooling all the filtrates with the peptides obtained before (Step 7).
9. Add 5 μg of fresh trypsin to the retentate again, and collect peptides by centrifugation at 14,000 × *g* for 15 min at room temperature. Add 30 μL of UB solution and centrifuge at 14,000 × *g* for 15 min at room temperature. Repeat this step three times. By combining all the filtrates (typical final volume around 250–300 μL), a significantly high metal–peptide global recovery was obtained [2].
10. After completing the digestion step, desalt and concentrate the digests by micro-solid phase extraction using OMIX tips (C_18_, 100 μL) as described the manufacturer, eluting the peptides in 50 μL of 70% acetonitrile and 0.1% formic acid. Evaporate the eluates to dryness in a centrifugal evaporator and reconstitute in 10 μL of buffer A for nLC–ESI-LTQ-MS/MS analysis, as described earlier in the Protocol A (step 15).
